# Robust and efficient COVID-19 detection techniques: A machine learning approach

**DOI:** 10.1371/journal.pone.0274538

**Published:** 2022-09-15

**Authors:** Md. Mahadi Hasan, Saba Binte Murtaz, Muhammad Usama Islam, Muhammad Jafar Sadeq, Jasim Uddin

**Affiliations:** 1 Department of Computer Science and Engineering, Asian University of Bangladesh, Ashulia, Dhaka, Bangladesh; 2 School of Computing and Informatics, University of Louisiana at Lafayette, Lafayette, Louisiana, United States of America; 3 Department of Applied Computing and Engineering, Cardiff School of Technologies, Cardiff Metropolitan University, Cardiff, Wales, United Kingdom; Hanyang University, REPUBLIC OF KOREA

## Abstract

The devastating impact of the Severe Acute Respiratory Syndrome-Coronavirus 2 (SARS-CoV-2) pandemic almost halted the global economy and is responsible for 6 million deaths with infection rates of over 524 million. With significant reservations, initially, the SARS-CoV-2 virus was suspected to be infected by and closely related to Bats. However, over the periods of learning and critical development of experimental evidence, it is found to have some similarities with several gene clusters and virus proteins identified in animal-human transmission. Despite this substantial evidence and learnings, there is limited exploration regarding the SARS-CoV-2 genome to putative microRNAs (miRNAs) in the virus life cycle. In this context, this paper presents a detection method of SARS-CoV-2 precursor-miRNAs (pre-miRNAs) that helps to identify a quick detection of specific ribonucleic acid (RNAs). The approach employs an artificial neural network and proposes a model that estimated accuracy of 98.24%. The sampling technique includes a random selection of highly unbalanced datasets for reducing class imbalance following the application of matriculation artificial neural network that includes accuracy curve, loss curve, and confusion matrix. The classical approach to machine learning is then compared with the model and its performance. The proposed approach would be beneficial in identifying the target regions of RNA and better recognising of SARS-CoV-2 genome sequence to design oligonucleotide-based drugs against the genetic structure of the virus.

## 1 Introduction

In late 2019, few patients were affected in pneumonia with a nescient symptoms known as respiratory syndrome coronavirus 2 (SARS-CoV-2) and later named as coronavirus disease 2019 (COVID-19). There is still in debate exactly where it grown up, but Epidemiological evidence shown that the virous spread from Wuhan, Hubei province from local sea food market. It is also confirmed the gene sequence was identified from Bats. According to World Health Organization (WHO), the virus was rapidly spread in worldwide over 6 million deaths and still growing continuously. The special attention of this virus was to spread very fast, adapt rapidly and affected with the infection of the major symptoms in fever, cough, muscle pain, and diarrhea. The similar symptoms can also be seen in mice, dogs, cats, camels, pigs, chickens, and bats [1].

SARS-Cov-2 are an encapsulated and carrying a positive sense of single-stranded RNA genome that belongs to subfamily Coronavirdiae. However, Micro ribonucleic acid (miRNAs) were initially identified in 1993 which controlls the timing of nematode Caenorhabditis elegans (Lee, Feinbaum and Ambros, 1993). Micro ribonucleic acid are literally quite small with an average 22 nucleotides in length available in plants, animals and some viruses including HSV, HIV-1, Dengue, Influenza, and SARS-COV-2 involved in biological processes [2].

According to the literature [3–7], micro ribonucleic acid exploration is crucial due to around 30 percentage of human genes are regulated by micro ribonucleic acid, influencing diverse biological processes including development, proliferation, cell differentiation, and metabolism across the various cell types.

Considering the various conditions to investigate how micro ribonucleic acid regulated under various conditions to comprehend the gene expression and disease phenotypes. The miRNAs can be produced by the most deoxyribonucleic acid (DNA) viruses, but miRNA expression is controversial in the case of RNA viruses because of their cytoplasmic replication and insufficient knowledge of the nuclear miRNA complex structure [8]. Therefore the exact mechanism of viral and cellular miRNAs are not adequately realised in viral infections. However, miRNAs have recently emerged as antiviral regulators of viral genes triggered by a coronavirus [9]. Gene silencing, miRNAs indeed can play a crucial role controlling the expression of transcription factors [10]. Therefore, using miRNAs to defeat COVID-19 can be groundbreaking.

MicroRNAs are consequent from pri-miRNAs more than 1000 nt in length and pri-miRNAs comprises a hairpin structure that realises from 60–120 nt [11]. The structural properties of these hairpins are characterised by pri-miRNAs thriving from the other RNA stem loop similar to the structures establish in the nucleus. In addition, the hairpin is cut off from the pri-miRNA to comprise the predecessor of miRNA (pre-miRNA) [12].

In order to detect miRNAs, it is important to distinguish pre-miRNAs from other hairpin-like sequences [13]. In order to the consideration of miRNA biogenesis and small interfering RNA design, the pre-miRNA prediction has lately become an exciting relevant area in miRNA research [14].

According to the literature [15], SARS-CoV-2 pre-miRNA identification desires the necessary equipment and real life oriented physical environment setup which resembles very expensive as laborious and burdensome. Instead, Machine learning (ML) can be an alternative approach in the way to lead in the research specifically in miRNA biology, and focusing on biomarkers for potential diseases [16].

The major issue with using machine learning to detect pre-miRNAs is that the number of well-known pre-miRNAs is typically few in comparison to the hundreds of thousands of candidate sequences in a genome, making this a high-class imbalanced classification challenge [17]. H. sapiens genome is an example that has 1710 well-known pre-miRNAs but over 400 million hairpin-like sequences resulting in a 1:28128 imbalance [18]. ML algorithms are generally representing with balanced data sets but in a supervised classifier, imbalanced data tend to produce a model biased towards the majority class, with low performance in the minority one yielding false positives [19]. Many computational approaches, including homologous search, comparative genomics, and machine learning, have been developed in recent decades to locate pre-miRNAs and to overcome the imbalanced miRNA (positive) and non-miRNA (negative) samples problems [20]. Specifically, some machine learning-based computational approaches such as DIANA-microT [21], TargetScan [22], TargetScanS [23], miRanda [24], mirSVR [25], RNA22 [26] and RNAhybird [27] have introduced a significant progress improving the performance of ML based pre-miRNA detection.

Performance is the main research gap in SARS-CoV-2 pre-miRNA identification. Other relevant limitations include artificial negative class. In this research, the RUNN-COV (Random Under sampling with Neural Network for COVID detection) models are presented. The proposed model performance was established, and their significant comparison, limitation and major challenges are introduced along with other existing methods. It is anticipated that this model will contribute to the fight against COVID-19 by improving its detection and subsequent study of the biological functions of SARS-CoV-2 pre-miRNAs, leading to effective and robust treatments.

One of the major contributions of our research lies in data visualization through exploratory data analysis and substantial research to understand the high-class imbalance problem and through investigation and experimentation finding the best technique which in our case is random undersampling to solve this tenacious problem thus decreasing the likelihood of overfitting and increasing classifier performance in the process. While the main contribution lies in fine-tuning the model performance, but associated experimentation’s of exploratory data analysis specifically t-distributed stochastic neighbor embedding (t-SNE) were investigated thoroughly to understand the data loss patterns as well as separation pattern between negative and positive data points which ultimately aided us in identifying a more robust, decision boundary that generated better model performance. All these exploratory data analysis mechanisms, aided us in selecting the best parameters and algorithms, which when fed into our own model produced a substantially superior performance outperforming several limitations discussed above. The final contribution of our research is performance comparison, where we compared our approach and results with existing literature and their performance that would aid the researchers in understanding the latest state of research in this field and where to go next.

To summarize, this paper presents a random undersampling technique that deals with the high-class imbalance problem. In addition, several techniques including correlation matrix, t-SNE are investigated for data loss visualization, data point visualization, and identifying hidden patterns. The RUNN-COV model has represented with an extraordinary result which compared to the other existing techniques and possible recommendation of their limitations. This paper also shows a performance comparison with other relevant machine learning models. Finally, the results were systematically evaluated using nine evaluation metrics.

The rest of the paper is organized as follows. Section 2 introduces the inspiration of the SAR-CoV-2 pre-miRNA identification. Section 3 presents a survey of the literature for using computational approaches in the COVID-19 and relevant miRNA context. Section 4 presents a description of the SARS-CoV-2 dataset. Section 5 presents sampling strategy, clustering analysis, and RUNN-COV model architecture. Section 6 presents a detailed evaluation strategy, performance analysis, and statistical investigation. Finally, the paper concluded in Section 7.

## 2 Motivation

SARS-CoV-2 has had a tremendous impact in the world, not just in terms of health care but also in others including agriculture and food security, economic and financial, educational, industrial, power and energy, oil market, employment, and environmental [28]. Therefore, select the effective ways to diagnose the infection to control the spread of COVID-19 and generate a better treatment prospects are crucial.

As mentioned above, MicroRNAs (miRNAs) are the most powerful regulators of gene expression that play a role in practically all forms of gene regulation. Cellular miRNAs can be applied as therapeutic options for COVID-19 [8] as well as many other viral infections, such as Dengue [29], Influenza [30], Human Immunodeficiency Virus (HIV) [31], Herpes Simplex Viruses [32], and Hepatitis C Virus [33]. Viruses are incapable of self-replication without the machinery and metabolism of a host cell. Consequently, viruses employ various tactics, one of them being the modification of host cell miRNA to their advantage [30]. A disorder in the organism’s internal environment is generally accompanied by aberrant miRNA production or secretion in the cells or blood, which has become the key indicator to recognize deadly diseases like cancers [34], diabetes [35], cardiovascular diseases [36], and virus-caused diseases [37]. MicroRNAs can also interfere with the heart [38] and lung [39] disease caused by COVID-19. Because of the discovery of this link, there may be a great benefit in targeting miRNA-interaction genes to treat COVID-19. Also, nanobased miRNA vaccines can be utilized as nasal spray or drops to activate the immune response in the respiratory tract, which is the common initial location for SARS-CoV-2 viral entrance [8].

Unfortunately, currently there is one Food and Drug Administration (FDA) approved antiviral drug, Veklury (Remdesivir), for the treatment of COVID-19 under Emergency Use Authorization (EUA) [40] along with treatments that are under research ranging from other anti-viral drugs to plasma therapy, vaccines and antibody drugs. In the inadequacy of COVID-19 treatments and vaccines, miRNA-based therapeutic approaches may be an intriguing option for regulating the SARs-CoV-2 replication.

One possible avenue of attack is the design and synthesis of oligonucleotides against the genetic structure of SARS-CoV-2 with the aim to impede its replication or to degrade its genome [9], This is similar to the possibility of designing therapeutic oligonucleotides on the basis of the human genome [41].

The proposed RUNN-COV model will assist to detect of SARs-CoV-2 and potentially many other relevant RNAs as of interest. The findings demonstrate that contemporary machine learning technologies can be used to assist in responding to public health emergencies by helping to discover the characteristics of any viral agent and in devising novel therapeutic approaches.

## 3 Related works

Being able to reliably test for SARS-CoV-2 is essential to stopping its spread. Several types of tests exist to identify SARS-CoV-2, varying in how rapidly they give results, how sensitive they are, and how often they can be performed [42]. The Nucleic Acid Amplification Test (NAAT) is a high-sensitivity, high-specificity viral diagnostic test for SARS-CoV-2 [43] that can identify more than one viral RNA gene and specify whether the infection is current or recent. Antigen tests, which are low cost and are able to provide results rapidly, can find the presence of a specific viral antigen [44].

Among ML methods, support vector machine (SVM) was used to classify real human pre-miRNAs from pseudo pre-miRNAs with 90% accuracy [13]. When feature extraction methods were employed, the accuracy improved to 94.83% [45]. However, it is unclear whether the use of pseudo pre-microRNAs approximates the real scenarios that will be faced by actual testing equipment.

Human pre-miRNA classification was also attempted using deep learning, and contrasted with other machine learning techniques such as naive Bayes classifiers, k-nearest neighbors and random forest [46]. The under-sampling approach was used to overcome the class imbalance problem, and the model outperformed traditional machine learning models.

Plant miRNA detection has also been demonstrated, achieving 97.54% identification accuracy [47]. Human mirtrons and canonical miRNAs have been classified using convolutional neural networks (CNN) and long short-term memory networks (LSTMN) with 94.3% accuracy and 92.5% F1 score [48]. Human miRNA classification for gene prediction was performed with results of 90.02% sensitivity and 97.28% specificity [49]. The SVM-based porcine pre-microRNAs prediction method was proposed by [50], achieving a prediction accuracy of 95.6%.

The majority pre-miRNAs detection models are SVM classifier-based [51, 52]. SVM was used to detect animals and plant miRNAs and pre-miRNAs detection methods [53, 54]. Rice pre-miRNAs detection was done using random forest, achieving prediction accuracy of 93.48% [55].

When performing machine learning, most algorithms require both positive and negative examples. Databases of positive examples are readily available, but negative examples are scarce, forcing researchers to employ tactics such as creating negative examples through various means. This has various problems, among which is that there is no guarantee that an example that has been generated to be negative is not actually an undiscovered positive example [56].

This problem of class imbalance in SARS-CoV-2 pre-miRNA detection was demonstrated using various algorithms such as one-class SVM (OC-SVM), deeSOM, and mirDNN [57]. The imbalance ratio in the dataset was varied from 1:50 to 1:200, with decreasing performance as imbalance ratio increased. At the best imbalance ratio of 1:50, OC-SVM, deeSOM, mirDNN achieved F1 scores of 39%, 51% and 74% respectively. The focal loss function [58] was used to handle class imbalance, where larger weights are given to the more difficult-to-classify examples so that the problem of imbalance is ameliorated. Albahri OS et al. [59] reviewed AI-driven COVID-19 detection and classification using medical images. The research challenges and critical gaps had highlighted by the authors. Albahri AS et al. [60] provided a systematic review of AI-based data mining and machine learning algorithms for detecting and diagnosing COVID-19. This study analyzed the nature of the application, algorithms evaluation methods, and accuracy for COVID-19.

The RUNN-COV method presented in this work attempts to overcome the class imbalance problem through undersampling rather than negative example generation or weight adjustment.

## 4 Dataset

The dataset was used based upon pre-miRNA detection using machine learning techniques (Bugnon et al., 2021). It was derived by applying various techniques the SARS-CoV-2 genome from National Center for Biotechnology Information (NCBI) Reference Sequence NC_045512.2, resulting in 569 pre-miRNA samples with 73 features. The dataset also included 999888 hairpin-like sequences from the human genome as the negative class. The dataset detailed can be found in [61] where the positive samples are identified labeled as 1 and the negative samples indicated as 0.

**Fig 1:**
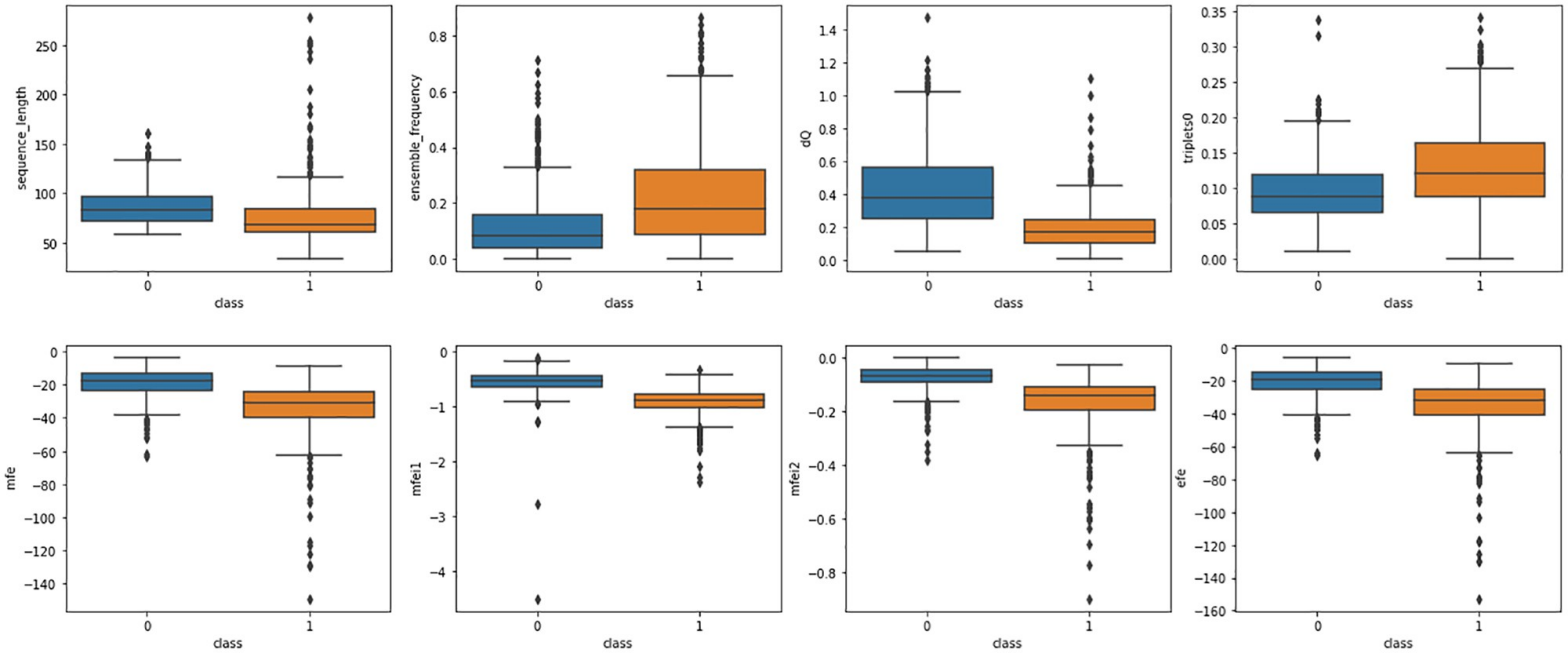
Box plots illustrate the distribution of numerical data with the pre-miRNA label class.

Pearson correlation was applied to the features in the dataset. It was found that many of the features are uncorrelated. Fig 1 shows the comparison between positive and negative samples. The central mark in the box indicates the median value, the edges of the box are the lower quartile and upper quartile values, and whiskers are goes to the minimum and maximum values. The outlier or single data point is depicted as the black dot. The sequence length, ensemble frequency, dQ, triplets0, mfe, mfei1, mfei2, etc are features of the dataset.

## 5 RUNN-COV model

### 5.1 Random undersampling

Random undersampling and oversampling are two techniques that are used to overcome the problem of class imbalance. Random oversampling involves duplicating the examples in the minority class, but this increases the likelihood of overfitting, decreases the classifier performance, and increases the computational effort [62]. Random undersampling instead removes examples randomly from the majority class, and was demonstrated experimentally to significantly improve classification performance [63]. Therefore, random undersampling was applied in this work to make the class ratio 1:1.

**Fig 2:**
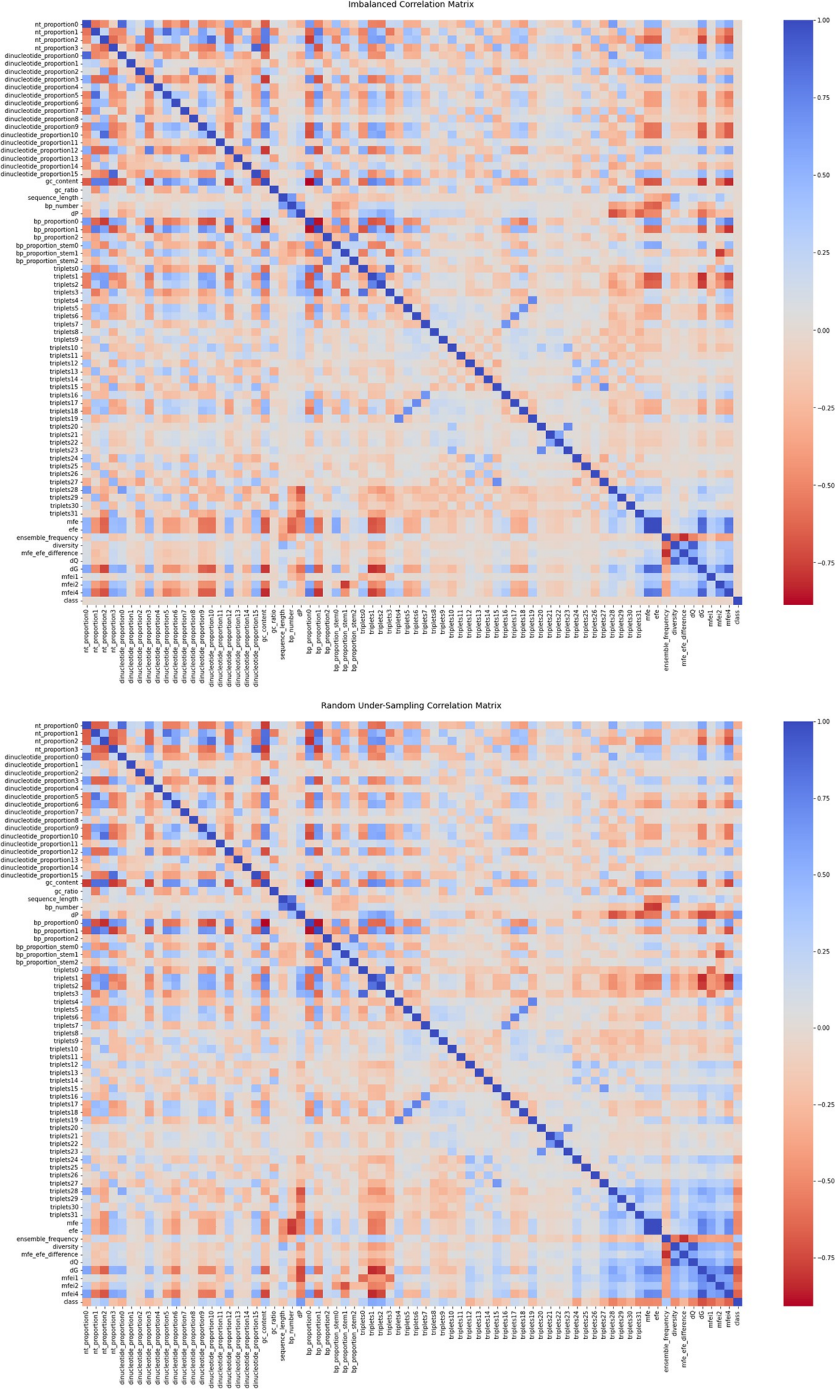
The heat map shows the patterns, similarities, associations, correlations and expression of pre-miRNA.

One concern with random undersampling is information loss when samples are removed. To demonstrate the effect of random undersampling, heatmaps of correlation matrices of the dataset before and after the procedure were taken. A random instance of from various runs of the experiment is given in Fig 2, where it can be seen visually that the heatmaps remain virtually the same before and after the random undersampling procedure. The variation of color depends on the intensity of the dataset feature. The correlation matrix before (top) and after (bottom) random undersampling.

After preparing the data through random undersampling, a visual representation is required to obtain a high-level understanding of the distribution of the positive and negative classes. In fact, visual understanding of high-dimensional data is crucial in many areas, such as the detailed analysis of single-cell datasets [64].

**Fig 3:**
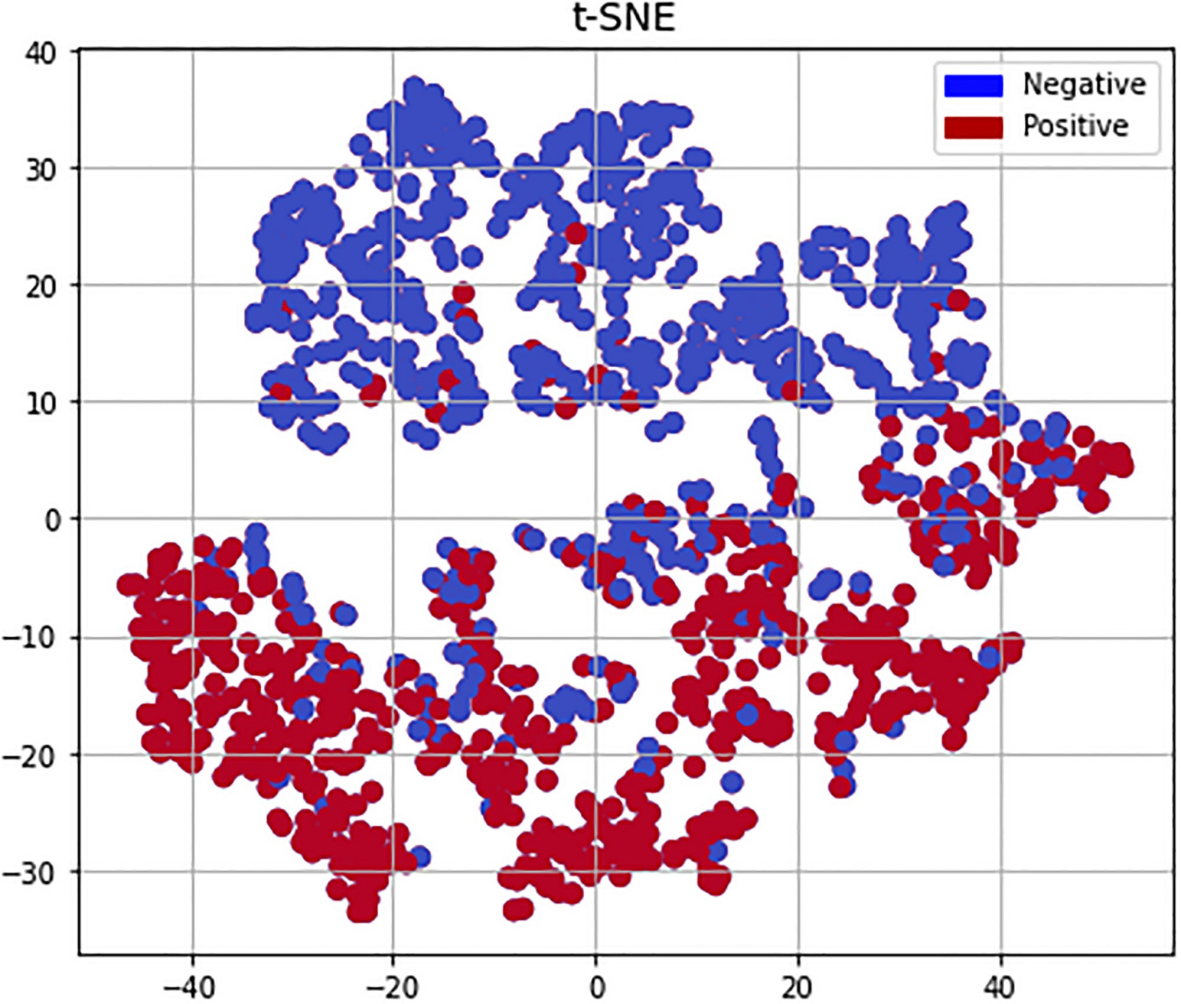
Overview of t-SNE-driven clustering analysis strategy. The landscape of the gene expression profiles represented high dimensional data in this two-dimensional map. The t-SNE projection shows the separation between positive and negative data points. In addition, the results of the pre-miRNAs cluster analysis are represented in two colors. The maroon dot indicates positive data point and the blue dot indicates the negation data point.

In this paper, t-distributed stochastic neighbor embedding (t-SNE), a nonlinear algorithm [65] for high dimensional data exploring, data point visualization, and identifying hidden patterns, was used to prepare a visual representation of the data as shown in Fig 3. The t-SNE was chosen because it outperforms a wide range of nonparametric visualization approaches [66]. It has become popular in the machine learning field because of its exceptional ability to generate two-dimensional (2D) maps from data with thousands of dimensions. The t-SNE is extremely flexible and can often identify structure where other dimensionality-reduction techniques cannot [67].

### 5.2 Neural network architecture

A neural network model was developed and applied to the data to determine if the positive and negative classes could be accurately identified. The model has 18 layers consisting of dense layers and batch normalization layers.

The rectified linear unit (ReLu) activation function was used in the dense layers, since it has various benefits such as computational simplicity, sparsity and linear behavior [68].

The batch normalization layers reduce training time and generalization error, minimize the over-fitting problem, increase stability, and smoothen the loss function [69]. The output layer uses the sigmoid function for binary classification.

The complete architecture has 1,128,994 parameters, of which 1,127,042 are trainable and 1,952 are non-trainable. The Adam optimizer was used to update network weights [70]. Other hyperparameter details include the use of the binary entropy loss function, 90 epochs, batch size 20, and data shuffling. The details of the neural network layers are shown in Table 1. This architecture was arrived at after extensive experimentation using different neural network architectures until one was found that produced high performance in identifying the examples of the dataset.

**Table 1:** Neural network structure.

Layer Number	Layer Type	Output Shape	Parameters
1	Dense	(None, 1024)	75776
2	Dense	(None, 512)	524800
3	Batch normalization	(None, 512)	2048
4	Dense	(None, 512)	262656
5	Dense	(None, 256)	131328
6	Batch normalization	(None, 256)	1024
7	Dense	(None, 256)	65792
8	Dense	(None, 128)	32896
9	Batch normalization	(None, 128)	512
10	Dense	(None, 128)	16512
11	Dense	(None, 64)	8256
12	Batch normalization	(None, 64)	256
13	Dense	(None, 64)	4160
14	Dense	(None, 32)	2080
15	Dense	(None, 16)	528
16	Batch normalization	(None, 16)	64
17	Dense	(None, 16)	272
18	Dense	(None, 2)	34

## 6 Results and discussion

### 6.1 Evaluation metrics

Various measures were chosen for evaluating RUNN-COV. The basic performance measures that are derived from true positive (TP), true negative (TN), false positive (FP) and false negative (FN): accuracy scores, along with precision, recall and F1 score, are shown in Eqs 1–4.

$$\begin{array}{c} Accuracy=\frac{TP+TN}{TP+TN+FP+FN} \end{array}$$ (1)

$$\begin{array}{c} Precision=\frac{TP}{TP+FP} \end{array}$$ (2)

$$\begin{array}{c} Recall=\frac{TP}{TP+FN} \end{array}$$ (3)

$$\begin{array}{c} F1\;Score=2\times \frac{Precision\times Recall}{Precision+Recall} \end{array}$$ (4)

Another more reliable derivative performance measure is the Matthews correlation coefficient (MCC), which only produces a high score if the prediction gained good results in TP, FN, TN, and FP [71].

$$\begin{array}{c} MCC=\frac{\left(TP\times TN)-(FP\times FN\right)}{\sqrt{\left(TP+FP)(TP+FN)(TN+FP)(TN+FN\right)}} \end{array}$$ (5)

The Cohen’s kappa (CK) is a robust statistic widely used to measure the algorithm performance [71].

$$\begin{array}{c} CK=\frac{Accuracy-Pe}{1-Pe} \end{array}$$ (6)

Hamming loss gives the fraction of all the labels that have been incorrectly identified.

$$\begin{array}{c} HL=\frac{1}{m}\sum_{i=1}^{m}\frac{|y_{i}\Delta y_{i}^{1}|}{Q} \end{array}$$ (7)

F-Beta (beta = 1.0) is the weighted harmonic mean between recall and precision.

**Fig 4:**
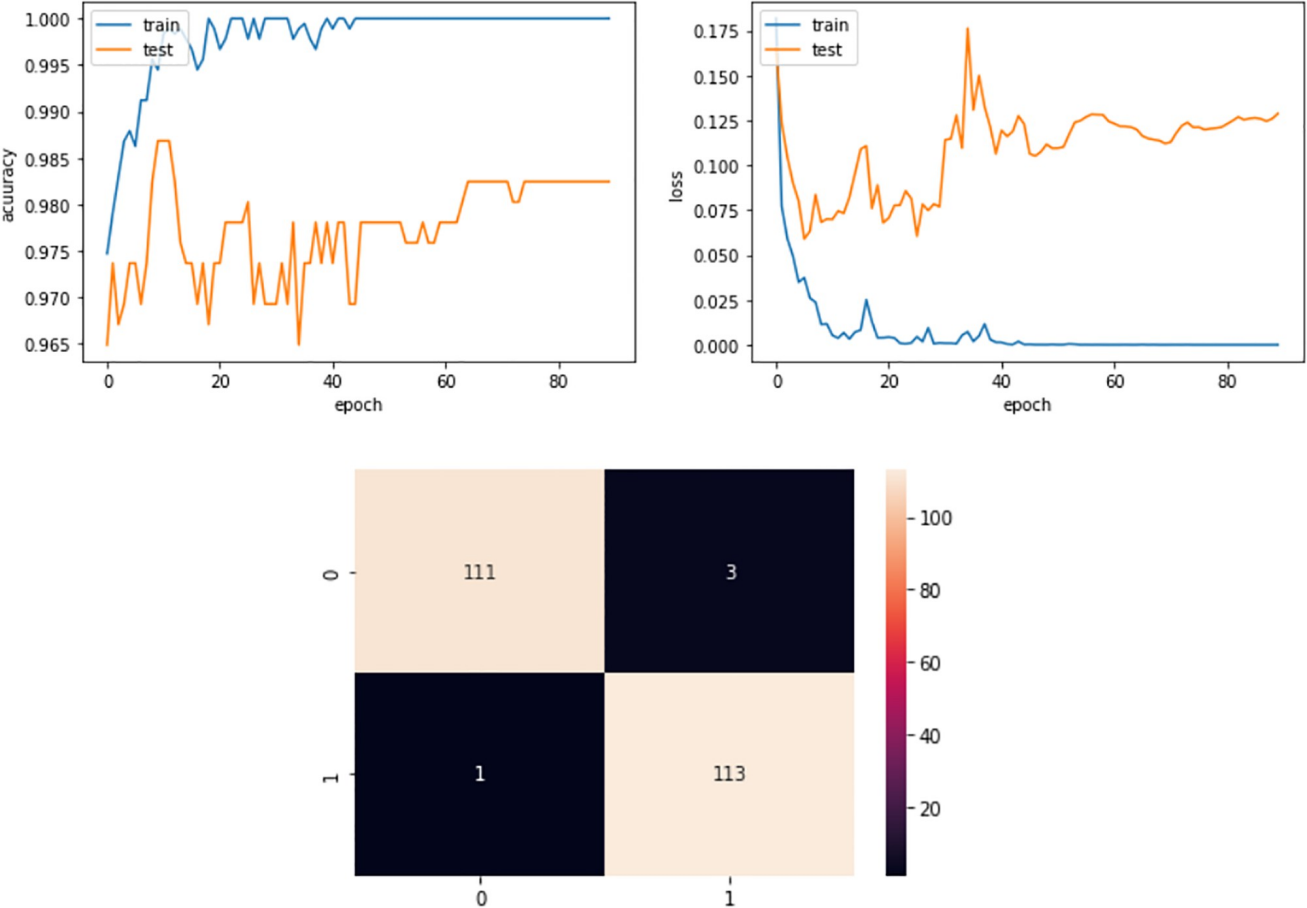
Accuracy curve, loss curve, and confusion matrix.

The dataset was split into 80% training data and 20% test data. Models were run with 90 epochs and the model scored well with the above performance metrics: accuracy 98.24%, Cohen’s kappa score 96.49%, Matthews correlation coefficient 96.50%, hamming loss 0.0175, precision 98%, recall 98%, f1-score 98%, area under the receiver operating characteristic (ROC AUC) score 98.24%, F-beta(beta = 1) score 98.26%. The accuracy curve, loss curve and confusion matrix are shown in Fig 4. The top left plot indicates the accuracy curve and the top-right plot the loss curve. Each plot x-axis shows time or epoch and y-axis learning or loss. The learning curve has improve over time. The bottom center plot illustrates the confusion matrix to observe the proposed model performance. The confusion matrix reflect the true positive rate at the top left corner, the false-positive rate at the top right corner, the false-negative rate at the bottom left corner, and the true-negative rate at the bottom right corner.

### 6.2 Comparison with other standard algorithms

The model’s performance was compared to the following popular machine learning algorithms.

- Logistic Regression is a popular algorithm used to used to solve classification problems [73], using gradient descent to reduce the cost. The logistic regression algorithm achieved 89.47% detection accuracy.
- The k-nearest-neighbours (KNN) classifies based on similarity of examples. It can help reduce the computational cost while maintaining classification accuracy [74]. The KNN classifier achieved 89.91% detection accuracy.
- Support Vector Machines (SVM) uses hyperplanes to separate classes [75]. The SVM classifier achieved 89.47% detection accuracy.
- Random Forest provides accurate results most of the time without hyper-parameter tuning [76]. The random forest creates several decision trees and merges them to get more accurate results. The random forest classifier achieved best 91.66% detection accuracy.

**Fig 5:**
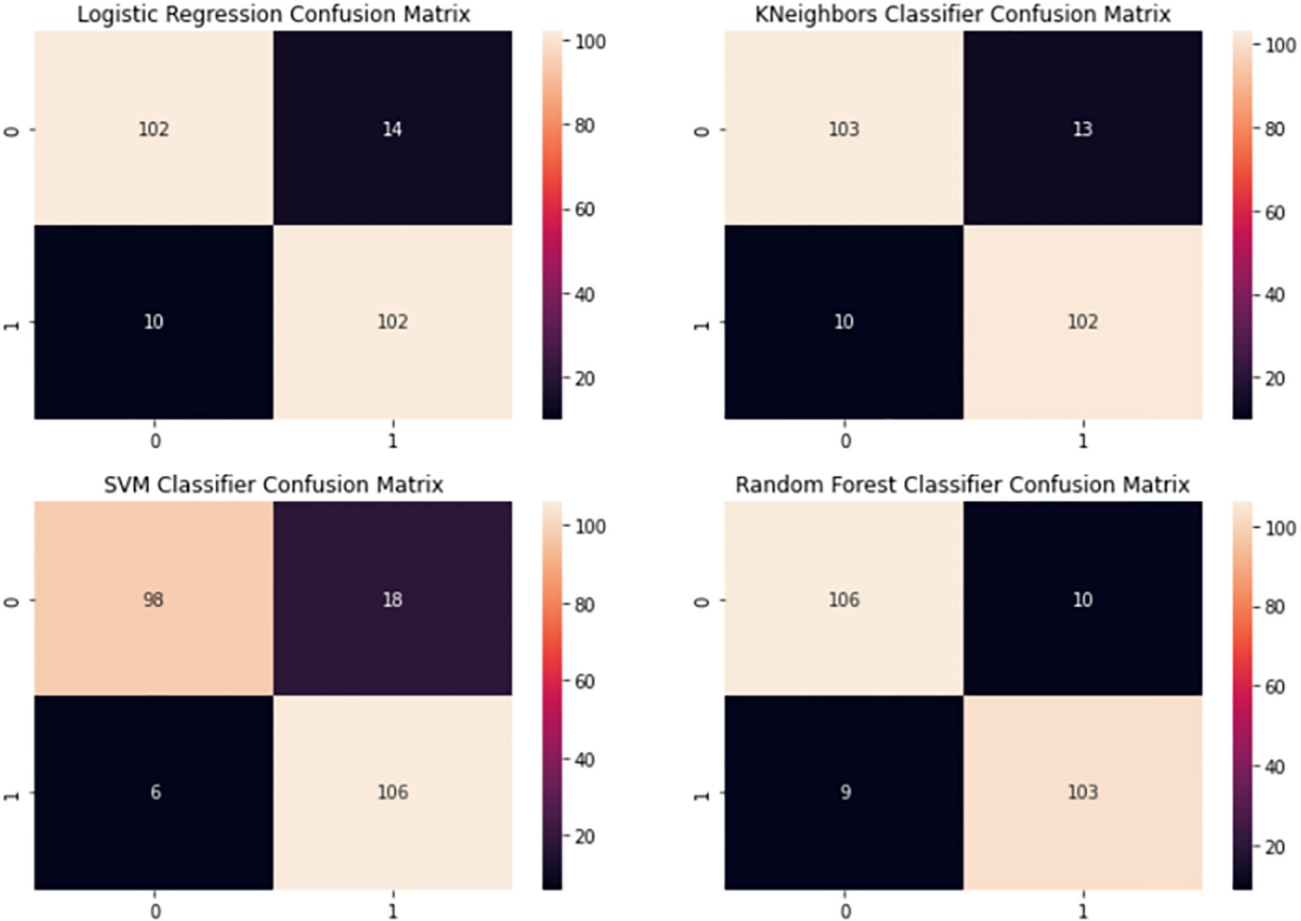
The confusion matrix shows the accuracy of traditional machine learning algorithms. The correctly classified data is reflected along the diagonal regions. The misclassified is reflected in the off-diagonal regions. Top-left plot logistic regression confusion matrix, top-right plot k-nearest-neighbors confusion matrix, bottom-left plot support vector machines confusion matrix, and bottom-right plot random forest confusion matrix.

By contrasting the confusion matrices (Figs 4 and 5) and from the performance metrics in Table 2, it was found that RUNN-COV performs better than these standard machine learning algorithms.

**Table 2:** Performance comparison with traditional ML models.

Model	MCC	Cohen’s kappa	Hamming Loss	Precision	Recall	F1 score	Accuracy	ROC AUC
**RUNN-COV**	**96.50%**	**96.49%**	**0.0175**	**97.41%**	**99.12%**	**98.26%**	**98.24%**	**98.24%**
RF	83.33%	83.33%	0.0833	91.15%	91.96%	91.55%	91.66%	91.67%
LR	79.00%	78.95%	0.1052	87.93%	91.07%	89.47%	89.47%	89.50%
SVM	79.41%	78.97%	0.1052	85.48%	94.64%	89.83%	89.47%	89.56%
KNN	79.85%	79.82%	0.1008	88.69%	91.07%	89.86%	89.91%	89.93%

**Fig 6:**
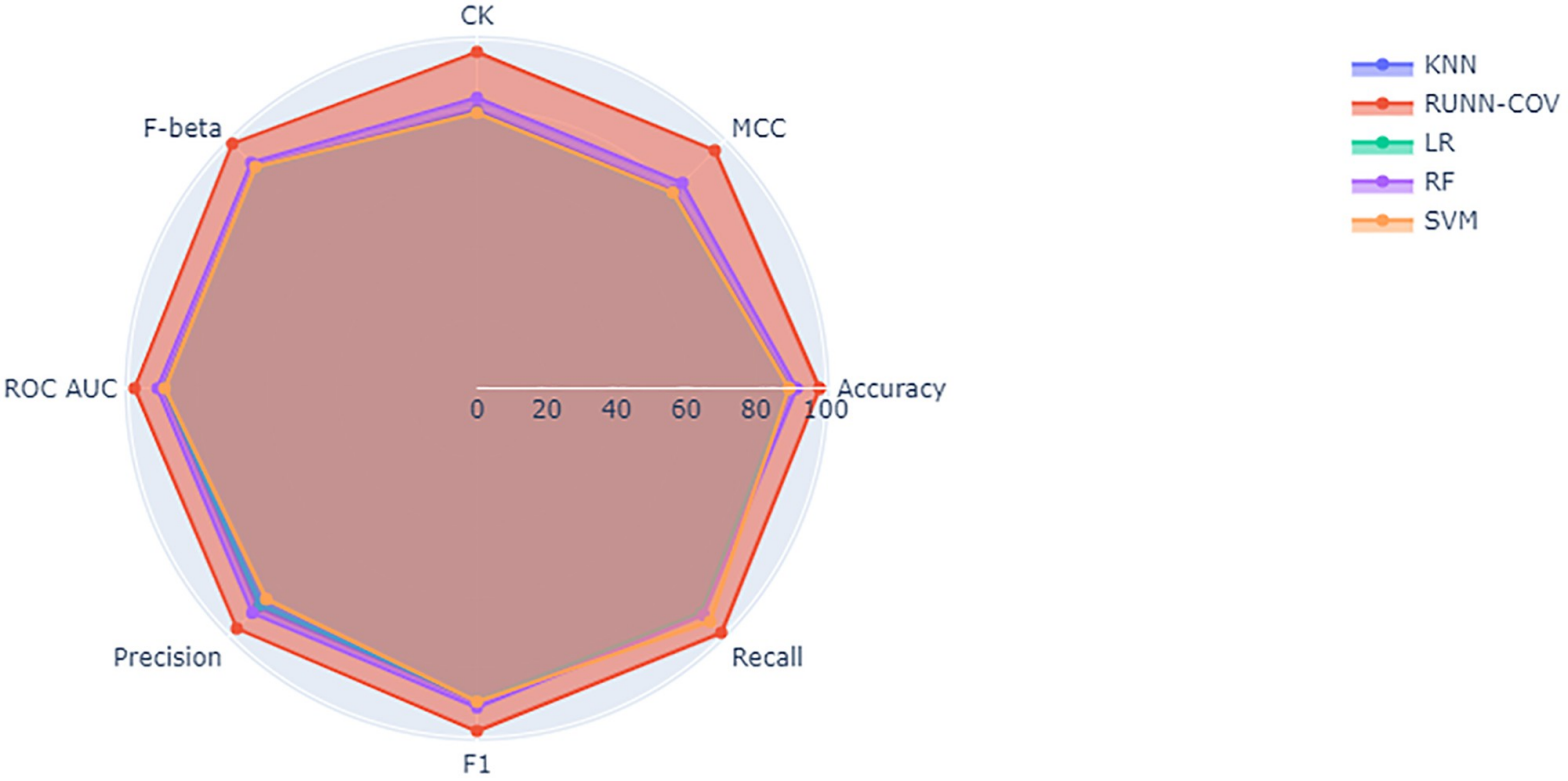
The radar chart illustrates the differences in performance metrics of KNN, RUNN-COV, logistic regression, random forest, and SVM algorithms. In this visual analysis, the different vertices show where each algorithm performs well and where each performs poorly.

Table 2 shows that RUNN-COV achieved detection accuracy 98.24%, faring better than logistic regression (89.47%), k-nearest neighbors (89.91%), support vector machines (89.47%), and random forest (91.66%). This is visually represented in Fig 6.

### 6.3 Performance comparison with existing approaches

Table 3 shows the comparison of RUNN-COV against various approaches in literature. Among them, the only one that used the same dataset achieved an accuracy of 51% [57] while RUNN-COV achieves 98.26% accuracy. RUNN-COV also has comparable results to other models that were run on different datasets.

**Table 3:** Performance comparison proposed model with relevant existing approaches.

Ref.	Models	MCC	Precision	Sensitivity/Recall	F1 score	Accuracy
[45]	MicroRNA-NHPred	89.65%	-	-	-	94.83%
[48]	CNN-LSTM	88.00%	-	94.80%	92.50%	94.30%
[48]	SVM	-	-	-	-	90.00%
[47]	PlantMirP2	-	-	96.75%	-	97.54%
[55]	Plantmirp-rice:	87.10%	-	87.91%	-	93.48%
[46]	DP-miRNA	-	-	97.30%	-	96.80%
[54]	PlantMiRNAPred	-	-	90.31%	-	92.06%
[57]	deeSOM	-	-	-	51%	-
[57]	OC-SVM	-	-	-	39%	-
**This paper**	**RUNN-COV**	**96.50%**	**97.41%**	**99.12%**	**98.26%**	**98.24%**

### 6.4 Statistical analysis

We used the statistical investigation to compare performance and novelty against previous studies. To compare the difference between the previous and proposed studies, we used a paired samples t-test. The statistical paired t-test is appropriate to compare statistical significance’s and differences [77]. To investigate performance used evaluation metrics including the F1 score and precision score.

$$\begin{array}{c} t=\frac{\bar{x}-\mu }{S/\surd N} \end{array}$$ (8)

In Eq 8, $\bar{x}$ is a sample mean *μ* is the constant for the population mean, *N* is the number of observations, and S/√N is the estimated standard error of the mean. We structured the following six null hypotheses:

x1H0: The deesom (1:50) Vs. RUNN-COV model performance have no significant difference.
x2H0: The OC-SVM (1:50) Vs. RUNN-COV model performance have no significant difference.
x3H0: The deesom (1:100) Vs. RUNN-COV model performance have no significant difference.
x4H0: The OC-SVM (1:100) Vs. RUNN-COV model performance have no significant difference.
x5H0: The deesom (1:200) Vs. RUNN-COV model performance have no significant difference.
x6H0: The OC-SVM (1:200) Vs. RUNN-COV model performance have no significant difference.

Table 4 shows the paired t-test results at 0.05 significance level and justifies the significant difference between the performance of previous studies and proposed studies. The paired t-test results that the p-value is less than 0.05 hence all six hypotheses x1H0, x2H0, x3H0, x4H0, x5H0, and x6H0 are rejected.

**Table 4:** RUNN-COV model against previous studies paired sample t-test (significance level of 0.05).

Model Pair	p-value
deesom (1:50) Vs. **RUNN-COV**	0.026
OC-SVM (1:50) Vs. **RUNN-COV**	0.021
deesom (1:100) Vs. **RUNN-COV**	0.027
OC-SVM (1:100) Vs. **RUNN-COV**	0.030
deesom (1:200) Vs. **RUNN-COV**	0.034
OC-SVM (1:200) Vs. **RUNN-COV**	0.024

With p-value less than 0.05 for all tests, with 95% confidence our model can be viewed as novel in nature and performs significantly better than existing approaches.

Table 5 displays our number of experiments, novelty, sampling strategy, variation of models and layers, as well as performance. Table 6 shows the p-values are less than 0.05, which indicates the significant difference between standard machine learning models against the proposed model.

**Table 5:** Number of experiments, sampling strategy, variation of models and layers, and performance against previous and proposed approaches.

Experiment	Strategy (Dataset)	Models	F1 score
Exp1	Without undersampling	RF	13.33%
Exp2	Without undersampling	LR	20.14%
Exp3	Without undersampling	SVM	05.21%
Exp4	Without undersampling	KNN	08.47%
Exp5	Without undersampling	Without Batch Normalization layers	47.17%
Exp6	Undersampling	RF	91.55%
Exp7	Undersampling	LR	89.47%
Exp8	Undersampling	SVM	89.83%
Exp9	Undersampling	KNN	89.86%
Exp10	Undersampling	Without Batch Normalization layers	92.10%
Exp11	Undersampling	With Dropout layers	92.10%
**Exp12**	**Undersampling**	**RUNN-COV**	**98.26%**
Previous literature	Ratio(1:50)	deeSOM	51.00%
Previous literature	Ratio(1:50)	OC-SVM	39.00%
Previous literature	Ratio(1:100)	deeSOM	42.00%
Previous literature	Ratio(1:100)	OC-SVM	28.00%
Previous literature	Ratio(1:200)	deeSOM	36.00%
Previous literature	Ratio(1:200)	OC-SVM	20.00%

**Table 6:** Paired sample t-test. (significance level of 0.05).

Model Pair	p-value
**RUNN-COV** Vs. Exp7	0.00040
**RUNN-COV** Vs. Exp8	0.00044
**RUNN-COV** Vs. Exp9	0.00094
**RUNN-COV** Vs. Exp10	0.00042

### 6.5 Discussion

In this research work, we have extensively explored the detection method of SARS-CoV-2 precursor-miRNAs through Artificial Neural networks. The initial dataset through experimentation provided a moderate performance which through dataset investigation, we unearthed existing class imbalance problem. Our research on solving high class imbalance problem, led us to further investigate solution manuals regarding class imbalancce problems and opted for random undersampling techniques. Similarly, As the dataset is noisy, it was handy for practitioners to cluster the dataset for which t-SNE was employed. t-SNE uncovers inexact contiguity in an basic high-dimensional complex, so clusters on the low-dimensional representation of the high-dimensional space maximize the probability that bordering data points will not be within the same cluster. Before employing t-SNE, we undertook the tradeoff that, t-SNE does not preserve distances nor density but preserves some form of nearest neighbours. t-SNE gave us 2D maps for the visualization of bias and variance from high-dimensional data. While the difference is subtle, but it affects any distance based algorithms at its core which led us to select distance-exclusionary algorithms that achieved extremely high- performance scores on various measures, outperforming traditional machine learning models and the other existing work on the same dataset. One might argue, why not use random oversampling instead of undersampling. We have investigated these as well albeit not reported in the results. We have realized that duplicating the instances in the minority class although solved class imbalance problem but it increased the likelihood of overfitting, as instances were increased gradually. Eventually, random oversampling contributed to a substantial decrease of classifier performance and increased computational error manifold that led us to stick to undersampling techniques.

An interesting instance of our neural network structure is susbsequent utilization of batch normalization. Although, using undersampling instead of oversampling minimized overfitting problem, in our experimentation, we still received anomaly through overfitting which was ultimately solved using batch normalization. Furthermore, this helped the network in reducing training time, smoothing the loss function and increased overall stability by reducing the generalization error. While we proposed the 18-layer exhaustive neural network, we have experimented the model with several variants of layers by including and excluding normalization and dropout layer as well. However, in our investigation, while including these two we have realized some interesting insights about neural networks in general. When using dropouts during training, activations are scaled to maintain the average after the dropout shift. However, the difference is not preserved. Traversing a non-linear slice translates this dispersion shift into an activation average shift, transitioning to the final linear projection slice. The final prediction is trained to fit the training time stats, so if dropout is off, it will fail during validation. This behavior is not an issue for tasks where only relative scaling of the output is important (such as softmax classification). In our case, if the output represents an absolute quantity, this leads to poor inference time performance. This architecture was arrived at after extensive experimentation using different neural network models.

The novelty of this research stands at experimentation of exploratory data analysis (EDA) including correlation matrix and t-distributed stochastic neighbor embedding. Efficient exploratory data analysis including data visual representation and data point analysis mechanisms aided us in selecting the best hyperparameters and models. RUNN-COV (Random Under sampling with Neural Network for COVID detection) models was presented based on previous EDA. Our designed model has produced a substantially superior performance which we have shown through experimentation. Statistical analysis was conducted against previous studies to understand the objective statistical significance of our research work that concluded our work is significant in nature.

While Tables 2 and 3 shows our model outperforming the previous literature as well as traditional machine learning approaches, the neural network models always hankers for space and time. The research can be continued further in evaluating this detection through more explainable mechanisms, which would aid us in excluding or including layers, and hyperparameters from architecture to make the model more robust and modular for daily usage. Semi-supervised learning such as active learning, sub-modular optimization, reinforcement learning can also be employed to attack the challenges again that were investigated in our research and reported in subsequent discussion to advance this field of research. The performance measures were also chosen to address the high class imbalance problem. We have observed that albeit having a greater accuracy, the accuracy metrics itself is not a good measure of performance in these highly imbalanced set of data. This lead us to experiment with precision and recall where precision provided us with an insight on how good the model was at predicting a specific type of target. Recall provides an insight on how many times the model detected a specific target. Overall, experimentation with performance measures such as MCC, F1- score were also added to the list for understanding the actual performance of our model.

## 7 Conclusion

COVID research necessitates an all-hands-on-deck strategy in order to eradicate the virus’s impact on the planet in a manner that is environmentally responsible. Among the various research domain specialists, computer scientists play a significant role in developing, analysing, and deploying cutting-edge research to continue the decent battle. In this study, a strategy is suggested that combines deep learning-based efficient pre-processing with neural network-inspired classification structures to identify SARS-CoV-2 pre-miRNAs effectively, therefore enhancing their performance. Our study demonstrates the effective processing and visualisation tools to generate insights, such as random undersampling, t-SNE, and correlation matrix, which gave insightful information that eventually increased the current research performance. The study examines various approaches to the class imbalance, adopted a random undersampling approaches and visualised data with t-SNE to generate better performances. Later, it is compared with existing approaches to classical Machine learning algorithms to provide an understanding of the contribution throughout the study.

This study has presented the RUNN-COV model, a neural network model with dense layers and batch normalisation layers for SARS-CoV-2 pre-miRNAs identification based on seventy-three features that enable the rapid detection of COVID. The model is comprised of using random undersampling techniques to the extremely imbalanced dataset in order to decrease class imbalance, followed by the use of a precisely designed artificial neural network. With Matthew’s correlation coefficient (MCC) score of 96.50 percent and an F1 score of 98.26 percent, the model outperformed typical machine learning models and other current work on the same dataset on a variety of other performance metrics. Additionally, the model performance is similar to that of other models that have been performed on distinct datasets.

It is believed that RUNN-COV will aid in the sequencing of the SARS-CoV-2 genome and the identification of target sites in an RNA in order to create oligonucleotide-based medicines against the genetic structure of the virus. Comparing this study with previous research, a comprehensive statistical analysis was undertaken to determine the objective statistical significance of the study.

Future work will include the development of a pre-miRNA detection technique based on raw RNA sequence data and the application of RUNN-COV to additional organisms’ datasets. Practitioners are also required to research to create more COVID-related data so that the issue of class imbalance may be resolved from the outset. In addition, prospects for study may be identified in the interpretation of results using explainable artificial intelligence, since the physicians, nurses, and patients are the most probable layperson consumers and stakeholders.

The whole research work including data visualization, exploratory data analysis, COVID detection task are available in github https://github.com/MdMahadiHasan1/SARS-CoV-2-pre-miRNA for repeatability and seamless replication.

## Supporting information

S1 File (DOCX)
