# Supplementary material for: Robust and efficient COVID-19 detection techniques: A machine learning approach
Source: PLoS One. 2022 Sep 15;17(9):e0274538. doi: 10.1371/journal.pone.0274538 (PMC9477266; doi:10.1371/journal.pone.0274538)
Supplement: S1 File — (DOCX) [file pone.0274538.s001.docx]

**Robust and Efficient COVID-19 Detection Techniques: A Machine Learning Approach**

# **Supporting Information**

This document presents an additional analysis of the dataset.

| **Index** | **Feature Name** | **Feature Type** |
| --- | --- | --- |
| 1 | nt_proportion0 | numerical |
| 2 | nt_proportion1 | numerical |
| 3 | nt_proportion2 | numerical |
| 4 | nt_proportion3 | numerical |
| 5 | dinucleotide_proportion0 | numerical |
| 6 | dinucleotide_proportion1 | numerical |
| 7 | dinucleotide_proportion2 | numerical |
| 8 | dinucleotide_proportion3 | numerical |
| 9 | dinucleotide_proportion4 | numerical |
| 10 | dinucleotide_proportion5 | numerical |
| 11 | dinucleotide_proportion6 | numerical |
| 12 | dinucleotide_proportion7 | numerical |
| 13 | dinucleotide_proportion8 | numerical |
| 14 | dinucleotide_proportion9 | numerical |
| 15 | dinucleotide_proportion10 | numerical |
| 16 | dinucleotide_proportion11 | numerical |
| 17 | dinucleotide_proportion12 | numerical |
| 18 | dinucleotide_proportion13 | numerical |
| 19 | dinucleotide_proportion14 | numerical |
| 20 | dinucleotide_proportion15 | numerical |
| 21 | gc_content | numerical |
| 22 | gc_ratio | numerical |
| 23 | sequence_length | numerical |
| 24 | bp_number | numerical |
| 25 | dP | numerical |
| 26 | bp_proportion0 | numerical |
| 27 | bp_proportion1 | numerical |
| 28 | bp_proportion2 | numerical |
| 29 | bp_proportion_stem0 | numerical |
| 30 | bp_proportion_stem1 | numerical |
| 31 | bp_proportion_stem2 | numerical |
| 32 | triplets0 | numerical |
| 33 | triplets1 | numerical |
| 34 | triplets2 | numerical |
| 35 | triplets3 | numerical |
| 36 | triplets4 | numerical |
| 37 | triplets5 | numerical |
| 38 | triplets6 | numerical |
| 39 | triplets7 | numerical |
| 40 | triplets8 | numerical |
| 41 | triplets9 | numerical |
| 42 | triplets10 | numerical |
| 43 | triplets11 | numerical |
| 44 | triplets12 | numerical |
| 45 | triplets13 | numerical |
| 46 | triplets14 | numerical |
| 47 | triplets15 | numerical |
| 48 | triplets16 | numerical |
| 49 | triplets17 | numerical |
| 50 | triplets18 | numerical |
| 51 | triplets19 | numerical |
| 52 | triplets20 | numerical |
| 53 | triplets21 | numerical |
| 54 | triplets22 | numerical |
| 55 | triplets23 | numerical |
| 56 | triplets24 | numerical |
| 57 | triplets25 | numerical |
| 58 | triplets26 | numerical |
| 59 | triplets27 | numerical |
| 60 | triplets28 | numerical |
| 61 | triplets29 | numerical |
| 62 | triplets30 | numerical |
| 63 | triplets31 | numerical |
| 64 | mfe | numerical |
| 65 | efe | numerical |
| 66 | ensemble_frequency | numerical |
| 67 | diversity | numerical |
| 68 | mfe_efe_difference | numerical |
| 69 | dQ | numerical |
| 70 | dG | numerical |
| 71 | mfei1 | numerical |
| 72 | mfei2 | numerical |
| 73 | mfei4 | numerical |

We used scatter plots which are the mainstay of statistical visualization. This depiction represents how variables in a dataset relate to each other.


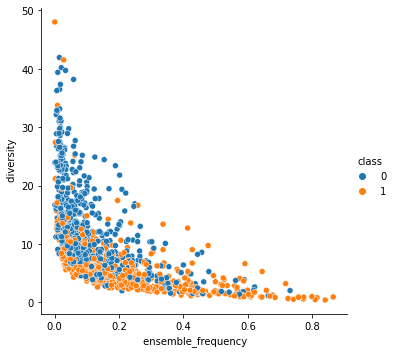


Fig 1: The horizontal axis represents ensemble frequency, the vertical axis represents diversity, and colors have illustrated according to the class.


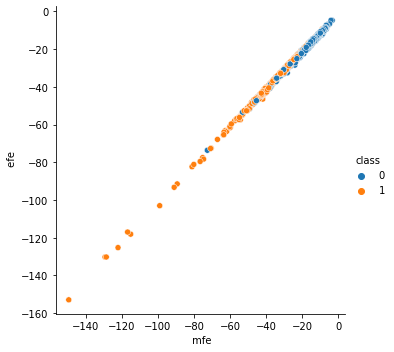


Fig 2: The horizontal axis represents mfe, the vertical axis represents efe, and colors have illustrated according to the class.


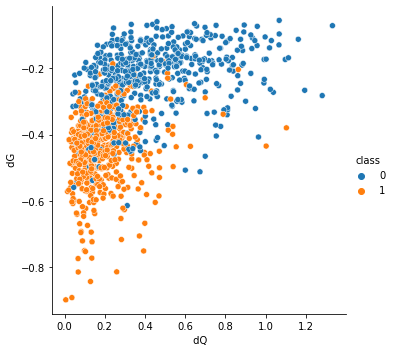


Fig 3: The horizontal axis represents dQ, the vertical axis represents dG, and colors have illustrated according to the class.


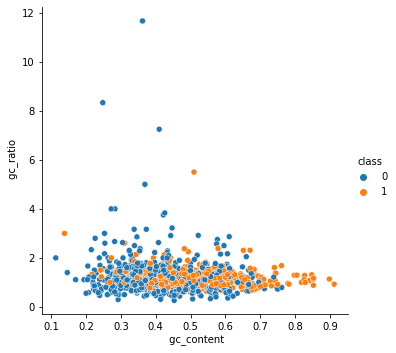


Fig 4: The horizontal axis represents gc_content, the vertical axis represents gc_ratio, and colors have illustrated according to the class.


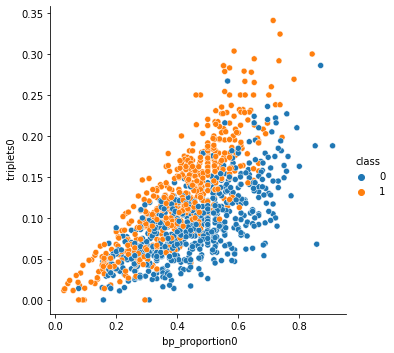


Fig 5: The horizontal axis represents bp_proportion0, the vertical axis represents triplets0, and colors have illustrated according to the class.


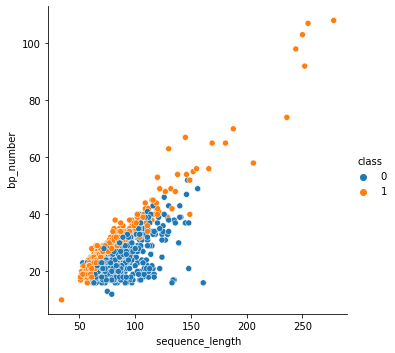


Fig 6: The horizontal axis represents sequence length, the vertical axis represents bp_number, and colors have illustrated according to the class.


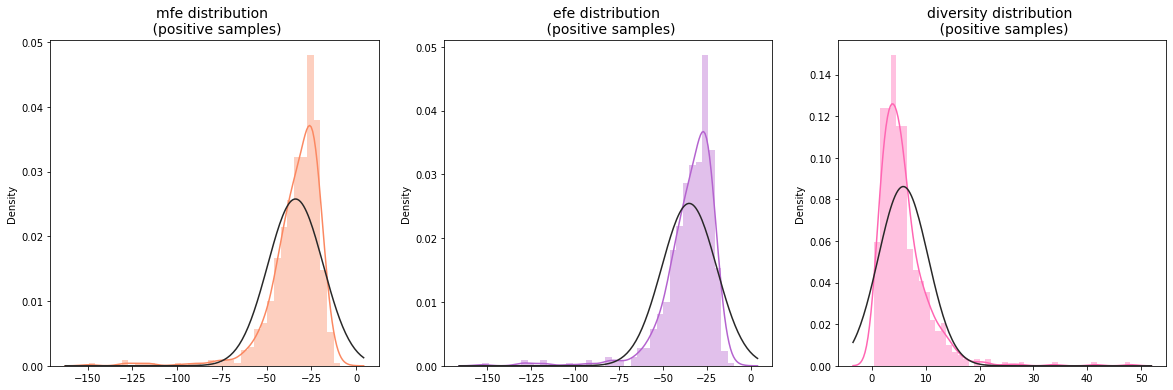


Fig 7: Shows the distribution of the dataset features.


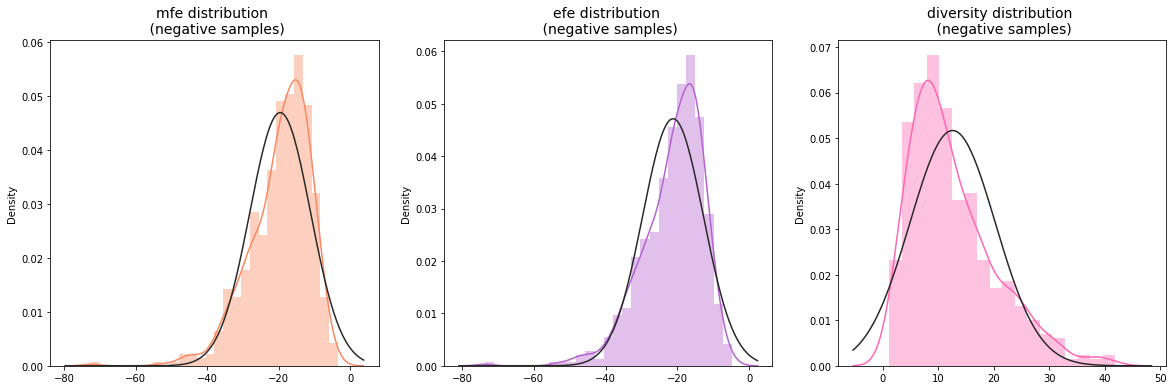


Fig 8: Shows the distribution of the dataset features
